# Citizen science to improve patient and public involvement in GUideline Implementation in oral health and DEntistry (the GUIDE platform)

**DOI:** 10.1111/hex.13921

**Published:** 2023-11-28

**Authors:** Annabel Hosie, Maria Firdaus, Jan Clarkson, Ekta Gupta, Lynn Laidlaw, Thomas Lamont, Margaret Mooney, Gillian Nevin, Craig Ramsay, Samantha Rutherford, Ana Margarida Sardo, Irene Soulsby, Derek Richards, Douglas Stirling, Michele West, Beatriz Goulao

**Affiliations:** ^1^ Health Services Research Unit University of Aberdeen Aberdeen UK; ^2^ Dental Health Services Research Unit, Dundee Dental School The University of Dundee Dundee UK; ^3^ Institute of Dentistry University of Aberdeen Aberdeen UK; ^4^ Patient partner UK; ^5^ Scottish Dental Clinical Effectiveness Programme (SDCEP) NHS Education for Scotland Edinburgh UK; ^6^ NHS Education for Scotland (Dental), Dundee Scotland UK; ^7^ Science Communication Unit University of the West of England Bristol UK

**Keywords:** citizen science, clinical guidelines, implementation of guidelines, oral health, patient and public engagement, patient and public involvement

## Abstract

**Background:**

Citizen science is a way to democratise science by involving groups of citizens in the research process. Clinical guidelines are used to improve practice, but their implementation can be limited. Involving patients and the public can enhance guideline implementation, but there is uncertainty about the best approaches to achieve this. Citizen science is a potential way to involve patients and the public in improving clinical guideline implementation. We aimed to explore the application of citizen science methods to involve patients and the public in the dissemination and implementation of clinical guidelines in oral health and dentistry.

**Methods:**

We developed GUIDE (GUideline Implementation in oral health and DEntistry), a citizen science online platform, using a participatory approach with researchers, oral health professionals, guideline developers and citizens. Recruitment was conducted exclusively online. The platform focused on prespecified challenges related to oral health assessment guidelines, and asked citizens to generate ideas, as well as vote and comment on other citizens' ideas to improve those challenges. Citizens also shared their views via surveys and two online synchronous group meetings. Data were collected on participant's demographics, platform engagement and experience of taking part. The most promising idea category was identified by an advisory group based on engagement, feasibility and relevance. We presented quantitative data using descriptive statistics and analysed qualitative data using inductive and deductive thematic analysis.

**Results:**

The platform was open for 6 months and we recruited 189 citizens, from which over 90 citizens actively engaged with the platform. Most citizens were over 34 years (64%), female (58%) and had a university degree (50%). They generated 128 ideas, 146 comments and 248 votes. The challenge that led to most engagement was related to prevention and oral health self‐care. To take this challenge forward, citizens generated a further 36 ideas to improve a pre‐existing National Health Service oral care prevention leaflet. Citizens discussed motivations to take part in the platform (understanding, values, self‐care), reasons to stay engaged (communication and feedback, outputs and impact, and relevance of topics discussed) and suggestions to improve future platforms.

**Conclusion:**

Citizen science is an effective approach to generate and prioritise ideas from a group of citizens to improve oral health and dental services. Prevention and oral health self‐care were of particular interest to citizens. More research is needed to ensure recruitment of a diverse group of citizens and to improve retention in citizen science projects.

**Patient or Public Contribution:**

This project was inherently conducted with the input of public partners (citizen scientists) in all key aspects of its conduct and interpretation. In addition, two public partners were part of the research team and contributed to the design of the project, as well as key decisions related to its conduct, analysis, interpretation and dissemination and are co‐authors of this manuscript.

## 1 INTRODUCTION

Citizen science refers to projects that involve nonprofessional ‘citizens’ directly in scientific research.^1^ Citizen science projects can have different goals focusing on the scientific output through activities such as data collection, or in opportunities to make science more democratic and responsive to the needs of citizens incorporating their personal experience.^2^ In healthcare, citizen science has been used to enable contributions in the development of health education resources, idea generation, evaluation of research and problem solving.^3^ Citizen science platforms, here defined as online platforms allowing asynchronous interaction between participants (from here onwards called citizens), have driven innovation in the UK's National Health Service (NHS) involving, for example, healthcare professionals' suggestions to improve services.^3^

Clinical guidelines provide evidence‐based guidance to healthcare practitioners, but their existence does not necessarily result in their implementation.^4^ Involving patients and the public in the development of clinical guidelines can impact their implementation,^5^ and research suggests that guidelines are more implementable when they include information to support patient involvement in decision‐making.^6^ The Scottish Dental Clinical Effectiveness Programme (SDCEP)^7^ produces oral health and dental clinical guidelines used in all UK nations. SDCEP involves oral health professionals in their clinical guideline development and implementation on an ongoing basis; however, patient input to support the production of patient materials or the implementation of clinical guidelines is limited. This is a common limitation of oral health clinical guidelines, with a recent scoping review of patient involvement in clinical guidelines finding no examples in the oral health field.^8^ Generally, patient involvement and engagement in clinical guidelines remain low despite multiple recommendations to do so,^9^, ^10^ with uncertainty about the best approaches to achieve this.^9^ To our knowledge, the involvement of patients or the public in clinical guideline development and specifically their dissemination and implementation through citizen science platforms has been underexplored; mostly, patient and public involvement in clinical guideline development has happened in‐person and has been exclusively facilitated by professionals.^8^

GUideline Implementation in DEntistry (GUIDE) was, to our knowledge, the first citizen science platform created to engage patients and the public in clinical guideline development.^8^ We focused on incorporating citizens' personal experiences to enhance clinical guidelines dissemination and implementation as outlined by Armstrong's framework.^11^ Using quantitative and qualitative methods, we aimed to explore the application of citizen science methods to involve patients and the public in clinical guideline development and, more specifically, the dissemination and implementation of clinical guidelines in oral health and dentistry.

## 2 METHODS

### 2.1 Study design

This is a participatory, longitudinal study using a citizen science approach via the GUIDE platform. Our study objectives were to: (1) collect and prioritise ideas from citizens to improve clinical guideline dissemination and implementation; (2) evaluate citizens' experiences of taking part in the platform. The project to develop and implement the GUIDE platform was conducted in collaboration with SDCEP and overseen by an advisory group including two public partners, four oral health professionals, three guideline developers and three research methodologists.

### 2.2 Development of the citizen science platform

GUIDE was an online platform hosted by a third‐party service, Crowdicity (https://www.medallia.com/crowdicity/). The general platform presented citizens with prespecified challenges, which included a topic area and questions to support their reflections. Challenges were defined as topic areas needing improvement with input from patients and this terminology was used across Crowdicity's citizen science platforms. It then invited citizens to generate suggestions to address the challenges. The platform also aimed to share evidence‐based recommendations with citizens to improve self‐care and oral health.

GUIDE focused on a single SDCEP clinical guidance topic: Oral Health Assessment and Review^12^ (i.e., dental check‐ups and preventive dentistry). The clinical guidance topic is aimed at oral health professionals and they were extensively consulted in its development. The oral health assessment guidance was originally published in 2011 and was selected to be explored in the platform because it aims to inform dental check‐ups, which are relevant to all patients. In addition, there is scope to improve adherence to these clinical guidelines,^13^ and an update of the clinical guidelines was being planned.

To select the most patient‐relevant recommendations within the Oral Health Assessment and Review guidance, we conducted a prioritisation and consensus exercise with GUIDE's advisory group. The exercise involved the following steps:

1. One researcher (B. G.) reviewed all clinical recommendations in the Oral Health Assessment and Review guidance and prepared a full list of recommendations.
2. All members of the advisory group were invited to read the full list and submit their priority recommendations independently (i.e., recommendations they thought were relevant to patients).
3. In an online group meeting, recommendations with any level of disagreement (i.e., not selected unanimously by the advisory group) were discussed until a consensus was reached about which recommendations should be prioritised.
4. Following this discussion, a final set of prioritised recommendations relevant to patients was agreed. B. G. grouped the prioritised recommendations into topic areas. The original topic areas explored were: Development of a personal care plan; prevention and oral health self‐care; oral health check‐ups; your oral health information.
5. The advisory group met again to generate ideas to describe each topic area as a challenge in the platform, including reflective questions.

### 2.3 Recruitment of citizens

**Figure 1:**
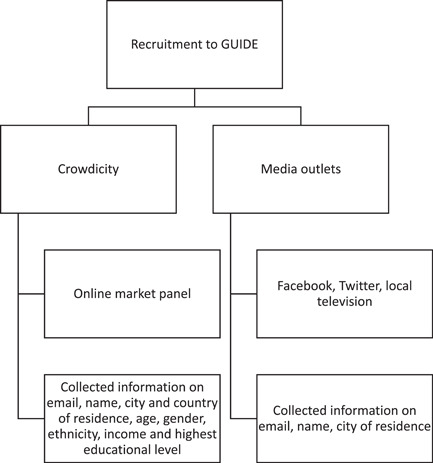
GUIDE (GUideline Implementation in DEntistry) recruitment routes and baseline information collected.

GUIDE citizens were recruited via two routes (summarised in Figure 1). The main source was via Crowdicity, using online market research panels with prespecified quotas reflecting the UK's distribution for all demographics collected. These quotas were set as an aim, but it was not mandatory to meet them if recruitment became unfeasible. Before joining the platform, potential citizens were informed of the expectations of membership (i.e., contributing weekly to the platform's discussions and generating ideas) and the generic topic of the platform (oral health). If they were interested in joining the platform, they were asked to provide basic demographic information as agreed by the advisory group (including country of residence, age, gender, ethnicity, income and highest educational level). They were then invited to join via email. Another recruitment route was via media outlets (including Facebook, Twitter and television, i.e., local news channels). If citizens joined the platform through these means, they were asked to provide their email address, name and city of residence. Inclusion criteria for joining the platform included living in the United Kingdom, English speaking, aged 18 or older and access to the internet.

### 2.4 Generation and prioritisation of ideas

There were various methods by which citizens could engage with the platform, including posting ideas related to the challenges, voting or discussing other ideas, sharing experiences, following other citizens in the platform, replying to surveys or attending synchronous online meetings. Data collection was embedded as part of the platform. Data relating to engagement with the platform were available at both participant and challenge level and included points (per participant based on their engagement with ideas and with the platform), votes (per idea), comments (per idea) and idea content.

To ensure the platform met its aim of sharing evidence‐based recommendations with citizens to improve self‐care and oral health, we included a blog function where we shared short texts written by the research team about self‐care and oral health. These texts were prepared according to the team's experience and expertise, as well as to ensure a connection with the platform discussions.

Citizens were provided with regular updates from the platform via newsletters. Following advice from Crowdicity's team to maintain engagement with the platform, researchers (A. H., M. F. and B. G.) engaged with citizens in the platform, mainly to incentivise and thank them for their participation. Following evidence suggesting gamification could enhance engagement of participants in citizen science platforms,^14^ citizens were awarded prespecified points each time they engaged with the platform, except when replying to surveys, as the host platform did not support this feature. Points were rewarded with badges (i.e., GUIDE champion) and were showcased on the platform's homepage in a ‘leader board’ (a ranking describing participant's points).

Prioritisation of ideas was agreed by the advisory group based on innovation, feasibility and relevance to SDCEP. To reach this decision, the following sources of information were considered:

1. The number of interactions with each original challenge (number of ideas, votes and comments).
2. Content analysis of the ideas generated and summaries of ideas collated into categories across challenges.
3. Feedback from the first synchronous online meeting with GUIDE citizens.

### 2.5 Evaluation of citizens' experience of taking part in the platform

We hosted two online meetings via Zoom and three short surveys with citizens and collected information on their experiences via open questions and group discussions. Questions focused on expectations/motivations when joining the platform; determinants of engagement (i.e., what prompted interacting with the challenges); what could encourage more participation and suggestions for platform improvements. Supporting Information S1: Material 1 provides examples of questions used in the surveys to evaluate citizens' experiences and is used in the online group discussions to facilitate the sessions.

### 2.6 Sample size

We aimed to recruit a minimum of 100 citizens, due to the platform's capacity and resources available. This is considered a medium‐scale citizen science project and above average in citizen science platforms related to health.^15^

### 2.7 Data analysis

Quantitative data were summarised using descriptive statistics. Demographics were presented as citizens who completed the recruitment survey and subsequently joined the platform versus those who did not join. Citizens recruited via social media did not complete the recruitment survey. To minimise missing data from these citizens, the following strategies were used: the country of residence of the citizens recruited via social media was determined by their city, displayed on their GUIDE profile; gender was determined by screen name or email address; and if no determinable name was available, they were classified as ‘unclear’. Age group, ethnicity, education and income were classified as ‘missing’. The platform automatically collected information on the number of ideas, comments, votes, participation in surveys or points received. We derived a variable summarising citizens' engagement as interaction with at least one of the following: idea, comment, vote, survey, online synchronous meeting or receiving points. This variable was presented using descriptive statistics.

Qualitative data collected related to ideas generated was gathered via direct downloads from the GUIDE platform and originally organised by the challenge. One researcher (B. G.) read through all ideas and discussions generated and then regrouped them per category in an inductive way, that is, based on the similarity of ideas discussed, performing a traditional content analysis.^16^ The categories were discussed with two researchers (A. H. and M. F.) actively involved in facilitating the platform with B. G., as well as with the advisory group.

Qualitative audio data collected in the online group meetings was captioned using the Microsoft Word dictate function and edited manually. The analysis was divided into the three main themes discussed with citizens: motivations, engagement and suggestions for platform improvement. We based our analysis process on Braun and Clarke's approach using both inductive and theoretical thematic analysis.^17^ Theoretical thematic analysis was based on frameworks for citizen science motivations^18^ and engagement.^19^ We started by applying those frameworks to our meeting data and adding any new themes that did not fit existing frameworks (inductive thematic analysis). The frameworks were agreed by A. H. and B. G., but the process of identifying new themes and developing a final coding framework was done independently and discussed to reach an agreement. Responses to open questions from the GUIDE surveys were reviewed case by case and their content was integrated into the existing themes. Quotes from the online group meeting transcript were organised into themes and used to illustrate them. Quantitative data summaries were prepared using Microsoft Excel and Stata 16. Qualitative data analysis was completed in Microsoft Word.

## 3 RESULTS

### 3.1 Citizens' recruitment

**Figure 2:**
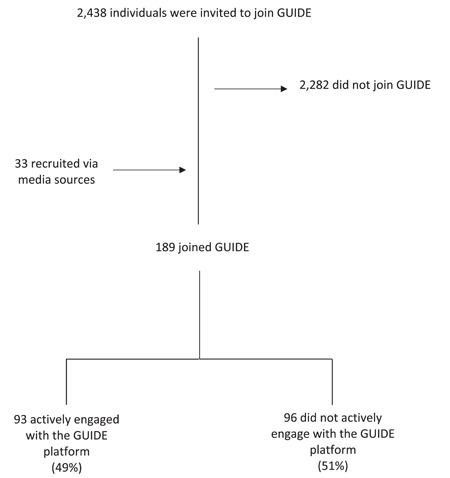
The number of individuals invited to join GUIDE (GUideline Implementation in DEntistry), the number who joined and the number who did not join.

The platform was open between September 2021 and March 2022, with recruitment between September 2021 and February 2022. A total of 2438 individuals were invited to join the GUIDE platform through online market research panels, 156 of whom joined (6.4% success rate) (Figure 2). A further 33 citizens were recruited via media sources; therefore, a total 189 citizens joined the GUIDE platform.

Supporting Information S1: Table S1 shows the demographics of GUIDE citizens who joined versus those who did not join GUIDE. Most GUIDE citizens were 35 years or older (64%), female (58%), White (72%), based in England (67%), with at least an undergraduate degree (50%) and earning more than £25,000 per year (56%). Citizens who replied to the initial survey but did not join GUIDE after invitation were similar and younger (58% were 25–44 years old).

Of the 189 citizens who joined the platform, 96 (51%) did not engage with any method and 93 (49%) engaged with at least one of the engagement methods. On average, citizens had five interactions with the platform (standard deviation = 20) with a minimum of zero and a maximum of 193 interactions. Overall, surveys were the most popular engagement method, with 49 out of 93 engagers (53%) completing at least one survey (Table 1). This was followed by creating an idea (46%), voting (29%) and commenting on others' ideas (23%). Fourteen per cent of citizens attended a synchronous online group meeting. At the end of this process, 29 citizens showed an interest in continuing their involvement in improving oral health services and research.

**Table 1:** Engagement and interest of GUIDE citizens.

	Frequency	%
*Citizens*	*N* = 189	
Citizens who engaged with at least one method	*N* = 93	49
Citizens who completed at least one survey	49	53
Citizens who created an idea in the platform	43	46
Citizens who were interested in continuing their involvement with the platform and/or oral health research	29	31
Citizens who voted in ideas	27	29
Citizens who commented on ideas	21	23
Citizens who attended at least one online meeting	13	14

Abbreviation: GUIDE, GUideline Implementation in DEntistry.

### 3.2 Idea collection and prioritisation (objective 1)

**Figure 3:**
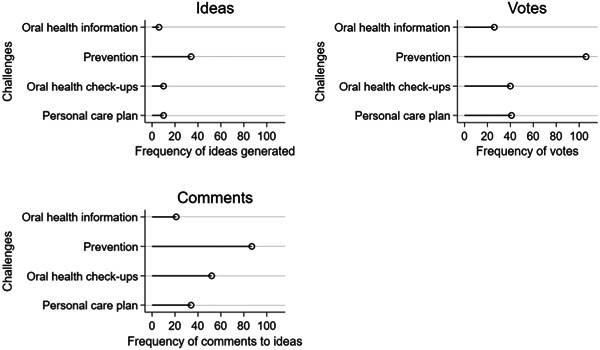
Frequency of different types of interaction (ideas, votes and challenges) with each original platform challenge.

While the platform was open, citizens contributed 128 ideas, 146 comments and 248 votes. On average, engaged citizens created 1.4 new ideas (varied from 0 to 31 unique ideas per participant, standard deviation = 4). Figure 3 illustrates the number of interactions in each original platform challenge: prevention was the most popular challenge with 34 ideas generated by citizens, 106 votes and 87 comments, followed by oral health check‐ups and personal care plans. Oral health information had the fewest ideas (*n* = 6), votes (*n* = 26) and comments (*n* = 21). These challenges were open for around 150 days. On average, the oral health self‐care and prevention challenge generated 0.20 ideas per day, all other challenges generated fewer ideas per day.

The content analysis of ideas suggested identified six different categories across challenges that could have a connection with oral health clinical guidelines:

1. *Oral health self‐care communication and questions* (e.g., creating a question and answer, NHS‐supported online platform, to discuss oral health self‐care with healthcare professionals; developing a public health campaign to share the most up‐to‐date advice in terms of brushing, interdental cleaning; access to demonstrations of good oral care).
2. *Ideas related to improving oral healthcare plans* (e.g., an app with reminders to floss and/or brush your teeth, personalised oral health plans in writing tailored to the patient's needs and delivered with an in‐depth conversation about how to deliver them).
3. *Frequency and type of contact between oral health professionals and patients* (e.g., ideas related to teledentistry and discussion about when this is appropriate, ideas for dentists to stay in touch between appointments for example by sending out standard surveys to check how the patient is doing and flag any problems).
4. *Ideas related to specific disease areas* (e.g., requesting more advice and information from the NHS about temporomandibular disorder based on participant's own experience of diagnosis and treatment).
5. *Ideas related to managing oral health anxiety* (e.g., keeping in touch with dentist virtually as a way to manage anxiety).
6. *Ideas related to sustainability* (e.g., suggestions about how patients can make their oral health routine more sustainable). Even though there was a high number of ideas generated across different topics, in general, ideas at this stage were broad and vague.

**Figure 4:**
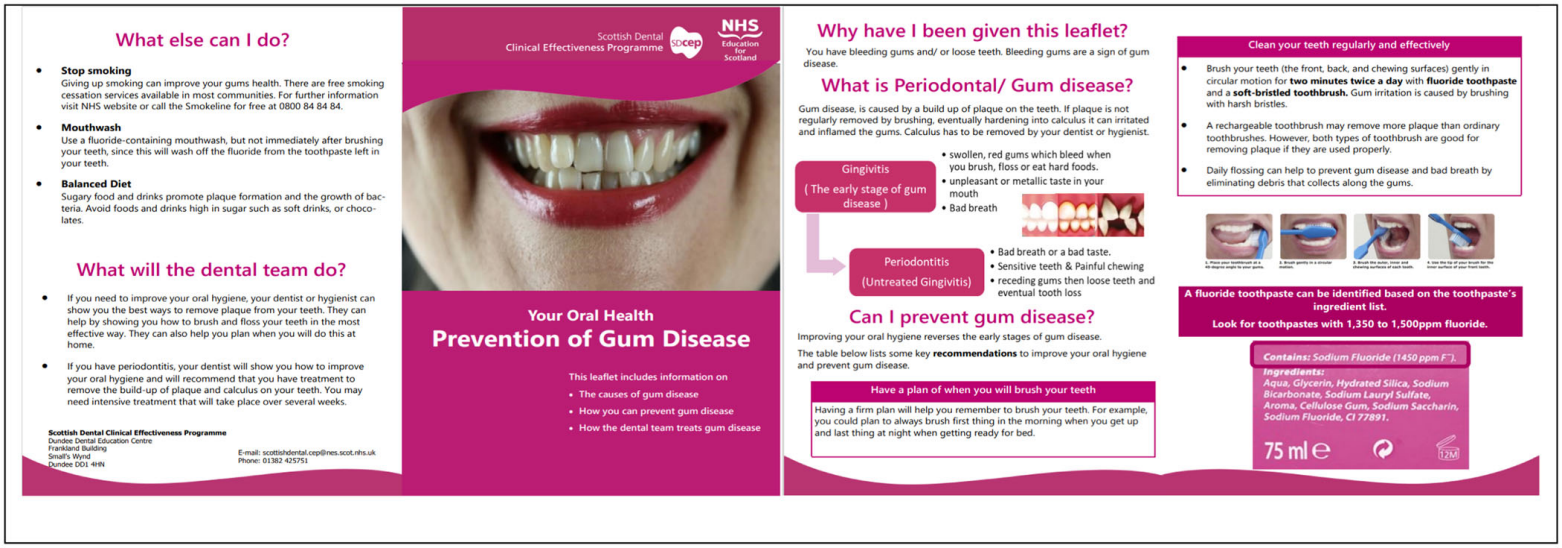
Updated version of National Health Service Leaflet.

A clear preference for discussion of self‐care and prevention of oral disease in the platform was confirmed by citizens in their first synchronous online group meeting. The advisory group met and was presented with the results of the content analysis, as well as the discussion from the synchronous meeting. Through discussion of the results as a group and considering feasibility of developing solutions that could be incorporated in new clinical guidelines, the advisory group reached consensus to prioritise ideas and solutions for prevention and oral health self‐care. For that reason, a final challenge to generate ideas to improve a pre‐existing NHS leaflet for self‐care was arranged (specifically, for prevention of gum disease^20^). The challenge was open for 19 days and generated on average over one idea per day with a total of 23 ideas. This was followed by a challenge with an updated leaflet asking for participant's feedback on the updated version and for suggestions for any further changes (‘The leaflet in this challenge has been edited to reflect YOUR suggestions and views. We would like to know if you think it does reflect your ideas, whether this version is better than the earlier version and whether there are aspects left to improve’). This was open for 13 days and resulted in one idea generated per day with a total of 13 ideas. Changes to the original leaflet included adding more images and a more realistic cover image; simplification of language; more focus on actions related to prevention including brushing and toothpaste choice. The overall feedback about the new leaflet (Figure 4) was positive, with citizens believing it reflected their comments and was an improved version of a self‐care leaflet aimed at patients.

### 3.3 Evaluation of citizens' experience of taking part in the platform (objective 2)

A combined 13 unique citizens attended the two online group meetings and a further six citizens answered survey questions related to their experience with the platform. In terms of their demographics (Supporting Information S1: Table S2), the majority were women and from England.

The results were divided into three main sections according to the topics discussed at the online group meeting: motivations, engagement and suggestions for improvement.

*Motivations to join the platform* focuses on reflections from citizens related to their decision to join the GUIDE platform. We found four themes when analysing motivations. They are: understanding, values, self‐care and concerns with oral healthcare services.

*Engagement with the platform* focuses on citizens' reflections on what promoted engagement in the platform. We found three themes in this analysis: feedback, communication and recognising contributions; impact and outputs; and relevance of topics discussed.

We illustrate with quotes each theme and subtheme (if applicable) within these topics in Table 2.

**Table 2:** Themes and subthemes were identified under the motivation and engagement topics discussed at the online group meetings and in the platform surveys.

Topic and themes	Subthemes if applicable	Illustrating quotes
*Topic: Motivations*		
Theme 1: Understanding Understanding summarises citizens' interest in seeking to learn more about oral health. We describe two subthemes within understanding: wanting to learn new things and trustworthy source of information.	*Subtheme 1: Wanting to learn new things*	‘I would like to hear about other people's dentistry experiences and how they deal with any problems they have and hope to learn from this and pick up any tips for maintaining a healthy mouth into older age’. (Participant 8, female, 65+, survey)
	*Subtheme 2: Finding trustworthy source of information*	‘I think the thing is as well, there's just so much information out there, you're not sure what is reliable and what is the truthful thing and sometimes you get on a site and you put in “oh I think I've got this problem” and then they try and sell you something or you get so far down the line and it's a consultation and you have to pay for it. So, you get a bit worried about that’. (Participant 4, female, online group meeting)
Theme 2: Values This theme summarises citizens' motivations related to expressing or acting on important values related to helping others or society in general. We describe two subthemes within values: helping other people and helping science.	*Subtheme 1: Helping other people*	‘I think self‐care is probably the one that would have the most impact on the most people…it probably is one of those things that would affect everyone or help everyone if we knew more about it’. (Participant 1, female, online group meeting)
	*Subtheme 2: Helping science*	‘I got involved with dental projects in Scotland, so I've been involved with the platform for a little while now’. (Participant 4, female, online group meeting)
Theme 3: Self‐care The most popular motivation for joining the GUIDE platform was to obtain advice to improve their own oral health self‐care.		‘… it would be useful to have somewhere to go for guidance on self‐care basically’. (Participant 6, male, 45–54, online group meeting)
Theme 4: Concerns about oral healthcare services Several citizens expressed concern about oral health services as their motivation for joining GUIDE.		‘I think my main recent concerns about dentistry is to do with COVID and its impact on the NHS’. (Participant 6, male, 45–54, online group meeting)
*Topic: Engagement with the platform*		
Theme 1: Feedback, communication and recognising citizens' contributions One participant expressed the importance of feedback for their own participation, with a lack of feedback discouraging their participation. Additionally, recognising their participation was a key contributor for engagement amongst online group meeting attendees		‘I think all ideas and comments should be acknowledged if only to indicate ‘that is interesting, but off topic’. (Participant 6, male, 45–54, survey)
Theme 2: Impact and outputs The online group meeting attendees identified that an important aspect to ensure their engagement with GUIDE was achieving tangible outcomes from their participation.		‘Going back to what you were saying about advice notes and that type of stuff, I think building towards that type of thing indicates we are making progress and I think you get sense of satisfaction from that’. (Participant 6, male, 45–54, online group meeting)
Theme 3: Relevance of topics discussed One online group meeting attendee noted that the relevance of the discussion could influence their participation.		‘I think relevance is obviously an issue in that some discussions are about specific conditions, some of which I've never heard of, and if you aren't affected by them then you're a lot less likely to engage with the matter’. (Participant 6, male, 45–54, online group meeting)

In terms of *improvement suggestions*, we collected a total of 21 improvements to the platform. The most common improvement suggestion related to requesting increased participant involvement with additional tasks. Additionally, citizens suggested they would like to receive more communication from the researchers and platform administrators. It was also suggested to improve the platform: the website design should be updated and simplified; a greater level of participant‐specific challenges, particularly relating to age groups; more recognition for their ideas and comments.

## 4 DISCUSSION

This is the first citizen science platform to incorporate citizens' views in oral health and dental clinical guidelines for dissemination and implementation. This was done through the development of patient‐facing materials (i.e., a new self‐care leaflet) to improve communication of clinical recommendations to patients. Over 90 citizens actively engaged with the GUIDE platform and one key priority was identified: the need for better and more trustworthy oral health self‐care communication. We identified citizens' key motivations to participate in the platform (understanding, values and self‐care) and their reasons to stay engaged (communication and feedback, outputs and impact, and relevance of topics).

Oral health self‐care was the most popular topic on the platform, with citizens showing an interest in learning more and supporting the development of resources. Self‐care information was clearly highly relevant to citizens on the platform, which is likely to be the reason that this topic received more attention. In addition, the coronavirus disease 2019 pandemic resulted in the closure of dental practices in the United Kingdom and subsequently patients have found it more difficult to access NHS oral health professionals.^21^, ^22^ Investing in self‐care was therefore seen as an unmet need. This priority is in line with recommendations to reshape preventive services in primary care dentistry.^23^, ^24^

GUIDE citizens presented similar motivations to join the platform as citizens in other citizen science projects, particularly those involved in health projects namely personal learning, making a difference and gaining health and well‐being benefits.^15^, ^18^, ^25^ This suggests the need to highlight potential health benefits and impact when recruiting to health‐related citizen science projects. On the other hand, the GUIDE citizens' reasons to stay engaged were more in line with patient and public involvement literature^26^, ^27^ and included frequent communication, acknowledgement and perceived impact. This information could inform future retention strategies in health‐related citizen science projects and the refinement of gamification or other engagement incentives.

Task‐oriented challenges proved to be more effective at keeping citizens engaged than general challenges asking for idea generation: challenges around developing and refining the leaflet had the highest and quickest engagement in the platform. This was expected, as the challenges were addressing citizens' interests, but they were also more task‐oriented than the original challenges. The task‐oriented approach provided a focus for citizens' ideas, as opposed to the broader (and vaguer) idea generation challenges that resulted in suggestions that could be difficult to put into practice as they did not have enough detail. This was a positive way to address a common barrier in citizen science projects of balancing the size of the project with the depth of the contributions from citizens.^15^ Our findings are in line with other research: a similar task‐oriented approach has been successfully tested with healthcare and other professionals generating ideas for a video related to maternity wards.^28^

GUIDE citizens improved and adapted a pre‐existing NHS self‐care leaflet, resulting in a final intervention endorsed by the citizens that could be adopted nationally in dental practices. The suggestions identified in our analysis are currently being used to inform an updated SDCEP clinical guidance topic (Prevention and Treatment of Periodontal Diseases in Primary Care), specifically its patient‐facing materials. This activity is in line with the examples provided by Armstrong et al. about how clinical guidelines can be improved through patient involvement in dissemination and implementation, namely by contributing to the development of summaries of guideline recommendations and patient‐facing materials.^11^ It also shows how citizen science can be used as an alternative to the commonly used in‐person methods to involve patients and the public in guideline development.^8^ Finally, it has the potential to address a top priority in oral health and dental research in facilitating communication between dental professionals and patients.^29^

Our platform was developed in a participatory way, reflecting views from researchers, guideline developers, oral health professionals and patients/citizens in our advisory group and following best practices for patient and public involvement.^27^ We collected ideas from citizens from all over the United Kingdom and explored their experiences of taking part in a citizen science project to improve healthcare, contributing to an underexplored field.^28^ The participatory nature of the project may increase the acceptability, uptake and impact of the ideas suggested to improve the NHS leaflet,^30^ but this remains untested; the ideas are currently informing the design of new leaflets for an updated SDCEP clinical guideline providing an opportunity to test these assumptions in the future.

GUIDE faced difficulties in recruiting a diverse sample of citizens, particularly regarding income and education, which is a commonly reported limitation in citizen science^1^ and patient and public involvement.^31^ Our sample's ethnicity distribution is closely aligned with the United Kingdom's distribution according to the Census 2021.^32^ However, the sample is overrepresented in middle‐aged categories (our sample's proportion of 35–64 years old is larger than the one observed in the UK population) at the expense of underrepresentation of younger (<34 years old) and older groups (>65 years old). We also had a higher proportion of women taking part when compared with the UK population, and in our sample, around 60% of citizens had level 4 or above education when compared with the United Kingdom's 34% according to the Census 2021.^32^ We attempted to maximise diversity in the platform by using recruitment quotas that reflected the UK's population; however, citizens approached in online market panels were also not reflective of the UK's population. Diversifying and tailoring recruitment strategies to reach underrepresented groups has been shown to improve the diversity of research participants.^33^ For example, working with oral health charities that can provide access to the internet, or working with oral health professionals that can provide support with the technology, is a potential way forward. Another possibility is to tailor citizen science platforms to better fit the needs of local or underrepresented communities as suggested by Pandya's inclusive citizen science framework.^34^ This could, in turn, result in citizen science platforms that are more inclusive and culturally sensitive. However, challenges about combining this approach with the current research and funding scheme demands remain and need to be further discussed.^35^ It is likely the current results do not reflect the views of those underrepresented in our sample, namely younger and older populations, and citizens with lower levels of education. It is imperative that future work explores this, as their needs are likely to differ from the ones captured in GUIDE, with well‐established inequalities in access to oral health services and treatment in England, especially by socioeconomic status.^36^

Only around half of the citizens recruited engaged with the platform, which is in line with findings reported from other online citizen science platforms.^37^, ^38^ Understanding why could help inform future strategies, but finding a way to reach out to disengaged citizens is notoriously difficult.^39^ One potential way forward would be to include questions at the recruitment survey in online marketing panels, for example, about motivations or expectations of joining. This information could be used to compare groups that go on to engage actively with the platform and those that do not, informing future strategies to enhance initial engagement. The platform did not include oral health professionals in the idea generation process for logistical reasons and because their views are already incorporated in the clinical guideline development process in a more systematic way. However, including healthcare professionals in an open dialogue with patients could improve the acceptability and uptake of resources developed, especially as there is evidence that views from the two groups regarding clinical guidelines might differ.^10^

## 5 CONCLUSION

The GUIDE platform facilitated an innovative approach to involve citizens (patients and the public) in clinical guideline dissemination and implementation by developing patient‐facing materials. These materials focused on a priority identified by the platform's citizens: the need for better self‐care information. The materials can be used to facilitate communication of clinical recommendations between dental professionals and patients. Future work should focus on maximising diversity and retention in citizen science platforms, contributing to inclusive patient and public involvement in clinical guideline dissemination and implementation.

## AUTHOR CONTRIBUTIONS

Beatriz Goulao, Craig Ramsay, Jan Clarkson, Thomas Lamont and Ana Margarida Sardo designed the study. Beatriz Goulao, Craig Ramsay, Jan Clarkson, Thomas Lamont, Ana Margarida Sardo, Douglas Stirling, Michele West, Samantha Rutherford, Lynn Laidlaw, Irene Soulsby, Gillian Nevin, Derek Richards, Margaret Mooney and Ekta Gupta contributed to the design of the platform and its challenges and decisions on data collection and prioritisation of an idea topic. Beatriz Goulao, Annabel Hosie, Maria Firdaus, Irene Soulsby and Margaret Mooney participated actively in the platform. Beatriz Goulao, Annabel Hosie and Maria Firdaus contributed to data collection and analysis. Annabel Hosie and Beatriz Goulao drafted the first version of the manuscript. All authors reviewed and commented on the manuscript before submission.

## CONFLICT OF INTEREST STATEMENT

The authors declare no conflict of interest.

## ETHICS STATEMENT

The project's protocol was ethically approved by the College of Life Sciences and Medicine Committee of the Ethical Review Board (CERB) (Application No. CERB/2021/5/2119) following relevant regulations and complying with the Declaration of Helsinki. All participants taking part provided informed consent. The protocol for this project was reviewed and approved by the School of Medicine, Medical Sciences and Nutrition Ethics Review Board (SERB) (Application No. CERB/2021/5/2119). Written informed consent (by accepting terms and conditions) was sought twice: initially, through the demographic data survey before joining the platform; and, subsequently, if participants decided to join the platform, consent was sought to collect their platform data for research purposes.

## Supporting information

Supporting information.

## Data Availability

The data that support the findings of this study are available on request from the corresponding author. The data are not publicly available due to privacy or ethical restrictions. The data sets used and/or analysed during the current study are available from the corresponding author upon reasonable request. The image used in Figure 1 is from https://guideoralhealth.crowdicity.com/.

## References

[1] Vohland K , Land‐Zandstra A , Ceccaroni L , eds. The Science of Citizen Science. Springer; 2021.

[2] Franzoni C , Poetz M , Sauermann H . Crowds, citizens, and science: a multi‐dimensional framework and agenda for future research. Ind Innov. 2022;29(2):251‐284. 10.1080/13662716.2021.1976627

[3] Parks S , D'Angelo C , Gunashekar S . Citizen Science: Generating Ideas and Exploring Consensus. THIS Institute; 2018:1‐16. https://www.thisinstitute.cam.ac.uk/using-citizen-science-to-generate-ideas-and-build-consensus-explored-in-new-report/

[4] Clarkson JE , Ramsay CR , Eccles MP , et al. The translation research in a dental setting (TRiaDS) programme protocol. Implement Sci. 2010;5(1):57. 20646275 10.1186/1748-5908-5-57 PMC2920875

[5] Armstrong MJ , Mullins CD , Gronseth GS , Gagliardi AR . Impact of patient involvement on clinical practice guideline development: a parallel group study. Implement Sci. 2018;13(1):55. 29661195 10.1186/s13012-018-0745-6 PMC5902835

[6] Gagliardi AR , Brouwers MC , Palda VA , Lemieux‐Charles L , Grimshaw JM . How can we improve guideline use? A conceptual framework of implementability. Implement Sci. 2011;6(1):26. 21426574 10.1186/1748-5908-6-26 PMC3072935

[7] Scottish Dental Clinical Effectiveness Programme. Accessed November 10, 2023. https://www.sdcep.org.uk/

[8] Bryant EA , Scott AM , Greenwood H , Thomas R . Patient and public involvement in the development of clinical practice guidelines: a scoping review. BMJ Open. 2022;12(9):e055428. 10.1136/bmjopen-2021-055428 PMC9528587 36171042

[9] Armstrong MJ , Bloom JA . Patient involvement in guidelines is poor five years after institute of Medicine standards: review of guideline methodologies. Res Involv Engagem. 2017;3(1):19. 29062544 10.1186/s40900-017-0070-2 PMC5623959

[10] Wu F , Burt J , Chowdhury T , et al. Specialty COPD care during COVID‐19: patient and clinician perspectives on remote delivery. BMJ Open Respir Res. 2021;8(1):e000817. 10.1136/bmjresp-2020-000817 PMC7797238 33414261

[11] Armstrong MJ , Rueda JD , Gronseth GS , Mullins CD . Framework for enhancing clinical practice guidelines through continuous patient engagement. Health Expect. 2017;20(1):3‐10. 27115476 10.1111/hex.12467 PMC5217879

[12] Oral Health Assessment and Review. Scottish Dental Clinical Effectiveness Programme. 2011. Accessed November 10, 2023. https://www.sdcep.org.uk/media/b1vlxume/sdcep-ohar-guidance-in-brief.pdf

[13] Beaton L , Clarkson J , Murray D , et al. Oral Health Assessment and Review: An Investigation of Current Practice and Beliefs. NHS Education for Scotland; 2019.

[14] Bowser A , Hansen D , He Y , et al. Using gamification to inspire new citizen science volunteers. ACM Int Conf Proceeding Ser. 2013;(2018):18‐25.

[15] Marks L , Laird Y , Trevena H , Smith BJ , Rowbotham S . A scoping review of citizen science approaches in chronic disease prevention. Front Public Health. 2022;10:1‐16. 10.3389/fpubh.2022.743348 PMC9125037 35615030

[16] Hsieh HF , Shannon SE . Three approaches to qualitative content analysis. Qual Health Res. 2005;15(9):1277‐1288. 16204405 10.1177/1049732305276687

[17] Braun V , Clarke V . Successful Qualitative Research: A Practical Guide for Beginners. Sage; 2013.

[18] Finkelstien MA . Intrinsic vs. extrinsic motivational orientations and the volunteer process. Pers Individ Dif. 2009;46(5‐6):653‐658. 10.1016/j.paid.2009.01.010

[19] Geoghegan H , Dyke A , Pateman R , West S , Everett G . Understanding motivations for citizen science. United Kingdom Environmental Observation Framework. 2016. Accessed September 15, 2023. https://www.ukeof.org.uk/resources/citizen-science-resources/MotivationsforCSREPORTFINALMay2016.pdf

[20] Your Oral Health: prevention of gum disease. Scottish Dental Clinical Effectiveness Programme. Accessed November 10, 2023. https://www.sdcep.org.uk/media/xqsl3tpw/sdcep-oral-hygiene-leaflet-for-patients.pdf

[21] Evans D , Mills I , Burns L , Bryce M , Hanks S . The dental workforce recruitment and retention crisis in the UK. Br Dent J. 2023;234(8):573‐577. 37117357 10.1038/s41415-023-5737-5 PMC10141865

[22] Duncan EM , Goulao B , Clarkson J , Young L , Ramsay CR . “You had to do something”: prescribing antibiotics in Scotland during the COVID‐19 pandemic restrictions and remobilisation. Br Dent J. 2021;231(11):695. 34815483 10.1038/s41415-021-3621-8 PMC8609985

[23] Clarkson JE , Pitts NB , Fee PA , et al. Examining the effectiveness of different dental recall strategies on maintenance of optimum oral health: the INTERVAL dental recalls randomised controlled trial. Br Dent J. 2021;230(4):236‐243. 33637927 10.1038/s41415-021-2612-0 PMC7908962

[24] Clarkson J , Ramsay C , Lamont T , et al. Examining the impact of oral hygiene advice and/or scale and polish on periodontal disease: the IQuaD cluster factorial randomised controlled trial. Br Dent J. 2021;230(4):229‐235. 33637926 10.1038/s41415-021-2662-3 PMC7908958

[25] Lehman E , Jepson R , McAteer J , Archibald D . What motivates volunteers to engage in health‐related citizen science initiatives? A case study of our outdoors. Int J Environ Res Public Health. 2020;17(19):6950. 32977509 10.3390/ijerph17196950 PMC7578925

[26] Mathie E . Patient involvement in health research can be enhanced by regular feedback. Health Service Journal. 2018. https://www.hsj.co.uk/comment/patient-involvement-in-health-research-can-be-enhanced-by-regular-feedback/7022294.article

[27] Baines R , Bradwell H , Edwards K , et al. Meaningful patient and public involvement in digital health innovation, implementation and evaluation: a systematic review. Health Expect. 2022;25(4):1232‐1245. 35526274 10.1111/hex.13506 PMC9327849

[28] van der Scheer JW , Woodward M , Ansari A , et al. How to specify healthcare process improvements collaboratively using rapid, remote consensus‐building: a framework and a case study of its application. BMC Med Res Methodol. 2021;21(1):103. 33975550 10.1186/s12874-021-01288-9 PMC8111055

[29] Oral and Dental Health Top 10. James Lind Alliance; 2018. Accessed September 15, 2023. https://www.jla.nihr.ac.uk/priority-setting-partnerships/oral-and-dental-health/

[30] Brett J , Staniszewska S , Mockford C , et al. Mapping the impact of patient and public involvement on health and social care research: a systematic review. Health Expect. 2014;17(5):637‐650. 22809132 10.1111/j.1369-7625.2012.00795.x PMC5060910

[31] Dawson S , Campbell SM , Giles SJ , Morris RL , Cheraghi‐Sohi S . Black and minority ethnic group involvement in health and social care research: a systematic review. Health Expect. 2018;21(1):3‐22. 28812330 10.1111/hex.12597 PMC5750731

[32] Office for National Statistics . *Census 2021*. 2022. https://www.ons.gov.uk/census/planningforcensus2021/ukcensusdata

[33] Hinton L , Kuberska K , Dakin F , et al. A qualitative study of the dynamics of access to remote antenatal care through the lens of candidacy. J Health Serv Res Policy. 2023;28(4):222‐232. 37084393 10.1177/13558196231165361 PMC10515462

[34] Pandya RE . A framework for engaging diverse communities in citizen science in the US. Front Ecol Environ. 2012;10(6):314‐317.

[35] Sorensen AE , Jordan RC , LaDeau SL , et al. Reflecting on efforts to design an inclusive citizen science project in West Baltimore. Citiz Sci Theory Pract. 2019;4(1):1‐12.

[36] Newton J , White S . Inequalities in Oral Health in England. Public Health England; 2021. https://assets.publishing.service.gov.uk/government/uploads/system/uploads/attachment_data/file/970380/Inequalities_in_oral_health_in_England.pdf

[37] Crall A , Kosmala M , Cheng R , et al. Volunteer recruitment and retention in online citizen science projects using marketing strategies: lessons from season spotter. J Sci Commun. 2017;16(1):A01.

[38] Aristeidou M , Herodotou C , Ballard HL , et al. Exploring the participation of young citizen scientists in scientific research: the case of inaturalist. PLoS One. 2021;16(1):e0245682. 10.1371/journal.pone.0245682 33465161 PMC7815142

[39] Newlands R , Duncan E , Presseau J , et al. Why trials lose participants: a multitrial investigation of participants' perspectives using the theoretical domains framework. J Clin Epidemiol. 2021;137:1‐13. 10.1016/j.jclinepi.2021.03.007 33727134

