# Supplementary material for: Citizen science to improve patient and public involvement in GUideline Implementation in oral health and DEntistry (the GUIDE platform)
Source: Health Expect. 2023 Nov 28;27(1):e13921. doi: 10.1111/hex.13921 (PMC10768863; doi:10.1111/hex.13921)
Supplement: Supplementary file 1 — Supporting information. [file HEX-27-e13921-s001.docx]

**Supplemental materials**

**Supplemental material 1. Examples of questions asked in evaluation surveys in the GUIDE platform & online group meetings**

Three surveys uploaded to the GUIDE platform were used to gather citizens’ views. Two

were uploaded before the online group meeting and one following it. A sample of the specific survey

questions used for analysis were:

1) What do you expect from taking part in the GUIDE platform eg: learn more about

oral health?

2) Whether you are interested in continuing your involvement with the platform or

prefer to leave us at this point, please let us know suggestions to improve the

platform in the future or any comments about your experience.

3) What were your motivations to join GUIDE? eg: to learn more about oral health or

to discuss a specific issue you have; concerns about lack of dental services etc.

4) What prompted you to engage with the challenges? eg: email reminders,

newsletter, points in the platform etc.

5) What could be improved with GUIDE? eg: more emails, more interaction and

involvement etc.

| **Online group discussions**  **The first online group discussion had the following structure:**   1. Welcome and overview 2. Introductions and general discussion of the early results of the platform (e.g. how many people joined, what ideas and challenges are the ones generating the most engagement) 3. Evaluation questions, focusing on three key questions:  - Why did you choose to take part in GUIDE? - What was surprising or unexpected about taking part? - [After showing the most popular challenges was related to oral health self-care and discussing whether this resonated with participants]: How can we use the platform to focus on discussions about self-care in an efficient way? What worked well so far and what can be improved? - Was the learning element of the platform useful? How can it be improved?  1. Conclusion   **The second online group discussion had the following structure:**   1. Welcome and overview 2. Self-care leaflet challenge and its changes after feedback from GUIDE citizens 3. Discussion  - Have we improved the leaflet? - What worked well for you in this activity? - How can we improve future activities and platforms? - Would you take part again? |
| --- |
|  |

**Table S1 - Demographics of citizens who joined the GUIDE platform and those who did not join**

|  |  | **Joined GUIDE**  **(N=189)**  **n(%)** | **Did not join GUIDE**  **(N=2,282)**  **n(%)** |
| --- | --- | --- | --- |
| **Age group** | 18-24 | 7 (4) | 269 (12) |
|  | 25-34 | 27 (14) | 711 (31) |
|  | 35-44 | 41 (22) | 611 (27) |
|  | 45-54 | 38 (20) | 394 (17) |
|  | 55-64 | 27 (14) | 192 (8) |
|  | 65+ | 16 (8) | 103 (5) |
|  | Missing | 33 (17) | 0 |
|  | Prefer not to say | 0 | 2 (0.09) |
| **Gender** | Male | 77 (41) | 1078 (47) |
|  | Female | 109 (58) | 1201 (53) |
|  | Unclear | 3 (2) | 0 |
|  | Other | 0 | 2 (0.09) |
|  | Prefer not to say | 0 | 1 (0.04) |
| **Ethnicity** | White | 137 (72) | 1968 (86) |
|  | Mixed/Multiple ethnic groups | 0 | 59 (3) |
|  | Asian | 14 (7) | 178 (8) |
|  | Black/African/  Caribbean/Black British | 2 (1) | 65 (3) |
|  | Other ethnic group | 3 (2) | 9 (0.4) |
|  | Prefer not to say | 0 | 3 (0.13) |
|  | Missing | 33 (17) | 0 |
| **Country** | England | 126 (67) | 1945 (85) |
|  | Scotland | 17 (9) | 186 (8) |
|  | Wales | 6 (3) | 103 (5) |
|  | Northern Ireland | 7 (4) | 48 (2) |
|  | Missing | 33 (17) | 0 |
| **Education** | Undergraduate degree | 57 (30) | 692 (30) |
|  | Postgraduate degree | 34 (18) | 436 (19) |
|  | Technical qualifications | 26 (14) | 515 (23) |
|  | Doctoral degree e.g. PhD | 4 (2) | 102 (4) |
|  | School leavers certificate | 35 (19) | 502 (22) |
|  | Missing | 33 (17) | 0 |
|  | Prefer not to say | 0 | 35 (2) |
| **Income p.a** | £0 | 1 (0.5) | 6 (0.3) |
|  | £1 - £9,999 | 10 (5) | 121 (5) |
|  | £10,000 - £24,999 | 35 (19) | 520 (23) |
|  | £25,000 - £49,999 | 58 (31) | 903 (40) |
|  | £50,000 - £74,999 | 31 (16) | 420 (18) |
|  | £75,000 - £99,999 | 12 (6) | 171 (7) |
|  | £100,000+ | 6 (3) | 87 (4) |
|  | Prefer not to say | 3 (2) | 54 (2) |
|  | Missing | 33 (17) | 0 |

**Table S2 – Demographics of online group meeting attendees and respondents to surveys**

|  | **Online group meeting attendees and survey respondents (N=19) - n(%)** |
| --- | --- |
| Age group |  |
| 35-44 | 2 (11) |
| 45-54 | 3 (16) |
| 55-64 | 2 (11) |
| 65+ | 1 (5) |
| Missing | 11 (58) |
| Gender |  |
| Male | 4 (21) |
| Female | 15 (79) |
| **Ethnicity** |  |
| White | 7 (37) |
| Asian | 1 (5) |
| Missing | 11 (58) |
| **Country** |  |
| England | 15 (79) |
| Scotland | 1 (5) |
| Missing | 3 (16) |
| **Education** |  |
| Undergraduate degree | 7 (37) |
| Postgraduate degree | 2 (11) |
| School leavers certificate | 3 (16) |
| Missing | 7 (37) |
| **Income p.a** |  |
| £1 - £9,999 | 1 (5) |
| £10,000 - £24,999 | 4 (21) |
| £25,000 - £49,999 | 4 (21) |
| £50,000 - £74,999 | 1 (5) |
| £75,000 - £99,999 | 2 (11) |
| Missing | 7 (37) |
